# Diversity and Distribution Patterns of Insects in Nigeria: A GBIF‐Based Appraisal

**DOI:** 10.1002/ece3.74090

**Published:** 2026-07-29

**Authors:** Emelie Obi, Mikaelison da Silva Lima, Kelechi Amadi, Maduamaka C. Abajue

**Affiliations:** ^1^ School of Science Dresden University of Technology Dresden Germany; ^2^ Department of Soil Zoology Senckenberg Museum of Natural History Görlitz Görlitz Germany; ^3^ Department of Biology University of Western Ontario London Ontario Canada; ^4^ Department of Biological and Environmental Science Georgia College and State University Milledgeville Georgia USA; ^5^ Department of Animal and Environmental Biology University of Port Harcourt Port Harcourt Nigeria

**Keywords:** arthropod, distribution, invertebrate, occurrence, sampling bias, species richness, West Africa

## Abstract

Insects provide essential ecosystem services and are key indicators of ecosystem health. Their diversity in Africa is estimated to be high, but they are poorly documented in many African countries, such as Nigeria. In this study, we used insect occurrence records from the Global Biodiversity Information Facility (GBIF) database to investigate and examine insect diversity and distribution patterns across Nigeria's major biomes. We analysed 49,854 records from the GBIF and found 19 orders, 305 families, 2515 genera and 5880 species. However, the richness estimator analysis suggests that current records only represent ~21% of the true insect diversity in Nigeria. We also show that diversity differs between biomes and follows a latitudinal pattern. Overall, our findings reveal geographic and taxonomic gaps, and highlight the need to prioritise under‐represented taxa and locations to improve biodiversity assessment and conservation planning. We suggest that the current state of Nigeria's insects reflects a dire situation that needs addressing through local and international collaboration facilitated by extensive government support.

## Graphical abstract

Insect diversity in Nigeria is high, with 5880 species. Diversity differs between biomes. Current diversity represents ~21 of the estimated ‘true’ diversity.

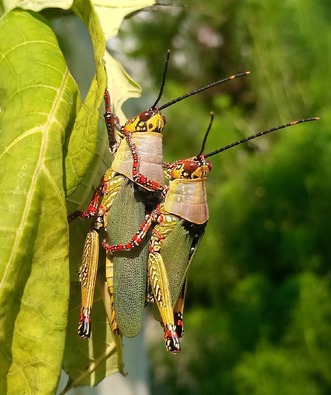

## SUMMARY IN NIGERIAN PIDGIN

Insects dey do plenty important work for nature, and dem be good sign to show whether environment still dey do well or not. Africa get plenty insects, but many African countries, like Nigeria, no know the kind of insects wey dem get. Na why we say make we check the number of insects wey dey for inside Naija. As we no get our own national insect record, we use one big data warehouse like that wey dem dey call GBIF (Global Biodiversity Information Facility). Wetin we see no be small thing o. Insects wey dey Naija plenty; we see say dem reach 5880 species. But as we carry calculation knack the thing, we see say even this 5880 species small o. Our calculation show us say the insects wey dey Naija fit actually be up to 12,000 but many places dey for Naija wey we never even check wether insects dey for there. This one mean say wetin we know about Naija insects small wella. For us to really know the real number of insects wey we get, we need urgent attention. We need to start dey study our environment and government too need to give us money for research. Because if we know the kind of insects wey we get, we go fit use dem well for our benefit, and also protect dem from wetin fit kill dem.

## 1 Introduction

Insects are among the most studied and described animal groups due to their overwhelming diversity and their importance to humans, animals and plants (Eggleton 2020; Aidoo et al. 2023). Globally, about 5.5 million insect species (dominated by beetles, butterflies and ants) have been estimated, but many (~80%) remain unknown (Stork et al. 2015; Stork 2018), especially in understudied regions such as Africa. Insect diversity in Africa (particularly Afrotropical Africa) is presumed to be high, with nearly 1 million species (Stork 2018). Nonetheless, country‐level records are lacking for many African countries. This is especially evident in Nigeria where, despite several reports of insects as pests (Allotey 1988; Orok et al. 1995; Ofuya et al. 2023), vectors (Okorie et al. 2011; Oguntomole et al. 2018; Okoro et al. 2023; Elosiuba et al. 2023) and food sources (Adeoye et al. 2014; Omuse et al. 2024; Idoko et al. 2026), insect diversity remains fragmented across isolated studies rather than summarised.

Nigeria is the most populous country in Africa, and is located on the Gulf of Guinea in the western part of the continent. It is the 14th‐largest country on the continent, with an area of ~923,769 km^2^, and lies between 4° N and 14° N of the equator and between 3° E and 15° E of the Greenwich meridian. Given its wide latitudinal extent, Nigeria is characterised by diverse climates that follow a latitudinal gradient (see Burgess et al. 2004). The southern part of the country receives the highest rainfall, while the northern part receives the lowest (Federal Ministry of Environment 2001; World Bank Group 2026). The biomes and vegetation also tend to follow this gradient (Federal Ministry of Environment 2001; Akpa et al. 2016; Jimoh et al. 2020; Okoro et al. 2025). For instance, the south is dominated by a humid forest that gradually transitions into different savannah biomes towards the north (Federal Ministry of Environment 2001; Akpa et al. 2016; Jimoh et al. 2020; Okoro et al. 2025). This provides habitats and microhabitats for different species of plants and animals (Federal Ministry of Environment 2001, 2025; Baker and Oates 2019; Osawaru et al. 2013; Yilangai et al. 2023), some of which are only endemic to Nigeria (Baker and Oates 2019; Borokini 2014). Reports on biodiversity in Nigeria indicate that there are ~22,000 invertebrate species (Federal Ministry of Environment 2001, 2025). However, this information is likely incomplete, as studies on Nigeria's biodiversity are generally fragmentary. For example, the closest representation of total insect diversity in the country appears when they are grouped with arthropods (Medler 1980; Federal Ministry of Environment 2001) or the recent checklist of Hymenoptera (Jimoh et al. 2024). Nonetheless, the overall composition and spatial patterns of insect diversity in Nigeria remain unknown.

Investigating insect diversity in a place like Nigeria can be challenging as there are no nationally established collections, and many areas remain under‐sampled and/or understudied (see Scholtz and Mansell 2017). In a situation like this, digitised occurrence data, such as from the Global Biodiversity Information Facility (GBIF), can provide valuable data. The GBIF is a global data platform funded by governments worldwide that provides open access to biodiversity data on all forms of life on Earth. It is a comprehensive database containing information from various sources, including literature, museum specimens and citizen records. In this study, we use GBIF occurrence records to present the first assessment of insect diversity in Nigeria with the aim of examining their taxonomic composition and spatial patterns. This is necessary for conservation strategies as insects continue to be threatened (Sánchez‐Bayo and Wyckhuys 2019; IUCN 2026) due to climate and anthropogenic changes.

## 2 Methods

### 2.1 Data Acquisition, Organisation and Summary

Insect occurrence records for Nigeria were sourced from the GBIF (GBIF.org 2026; https://www.gbif.org). Records were filtered to retain only valid binomial species names, excluding entries without species‐level identification or with ambiguous designations (e.g., ‘sp.’ or NA). The final dataset included 49,855 occurrence records and was summarised at the levels of order, family, genus and species using the R packages ‘dplyr’ and ‘tidyr’ (Wickham et al. 2024, 2026). The ten most species‐rich families and genera were visualised to highlight dominant taxonomic groups; similarly, we visualised the ten species with the highest occurrence records.

### 2.2 Data Preparation for Species Accumulation and Spatial Analysis

To allow us to perform spatial analyses, occurrence records were aggregated to 0.5° × 0.5° grid cells across Nigeria, following established methods for large‐scale biodiversity assessments (e.g., Gomes et al. 2018; Scheiter et al. 2024; Jenkins et al. 2024). This method reduces spatial redundancy while maintaining substantial resolution in large occurrence‐based datasets. Records lacking geographic coordinates or outside Nigeria's boundaries were excluded through spatial filtering using the R packages ‘sf’ (Pebesma 2018; Pebesma and Bivand 2023), ‘rnaturalearthdata’ (South et al. 2024) and ‘rnaturalearth’ (Massicotte and South 2026). The final dataset included 256 grid cells (hereafter ‘sites’), from which a presence–absence matrix of species occurrences was constructed. To compare species across ecological regions, each site was assigned to one of four major biomes in Nigeria (i.e., Sudan‐Sahel savannah, Guinea savannah, derived savannah and humid forest) roughly based on established vegetation classifications (Akpa et al. 2016; Jimoh et al. 2020; Okoro et al. 2025).

### 2.3 Data Analysis

We performed a species accumulation analysis on the presence–absence matrix to examine how species discovery increases with sampling effort (sites). To do this, we used the {specaccum} function in ‘vegan’ (Oksanen et al. 2024) and randomised the calculation with 1000 permutations. Further, we calculated estimated richness using the {poolaccum} function in ‘vegan’ as well. Estimated richness was calculated at both national and biome levels. We used the estimator, Chao2, as it is recommended for incidence‐based data (Chao 1987; Chao and Colwell 2017; Chao et al. 2017). Finally, we examined geographic variation in insect diversity; species richness was mapped by aggregating species counts within each grid cell. This was implemented with the packages ‘sf’, ‘rnaturalearthdata’, ‘rnaturalearth’ and plotted with ‘ggspatial’ (Dunnington 2025). All analyses were made in R (R Core Team 2024), and figures were made with the ‘ggplot2’ package (Wickham 2016).

## 3 Results

**FIGURE 1:**
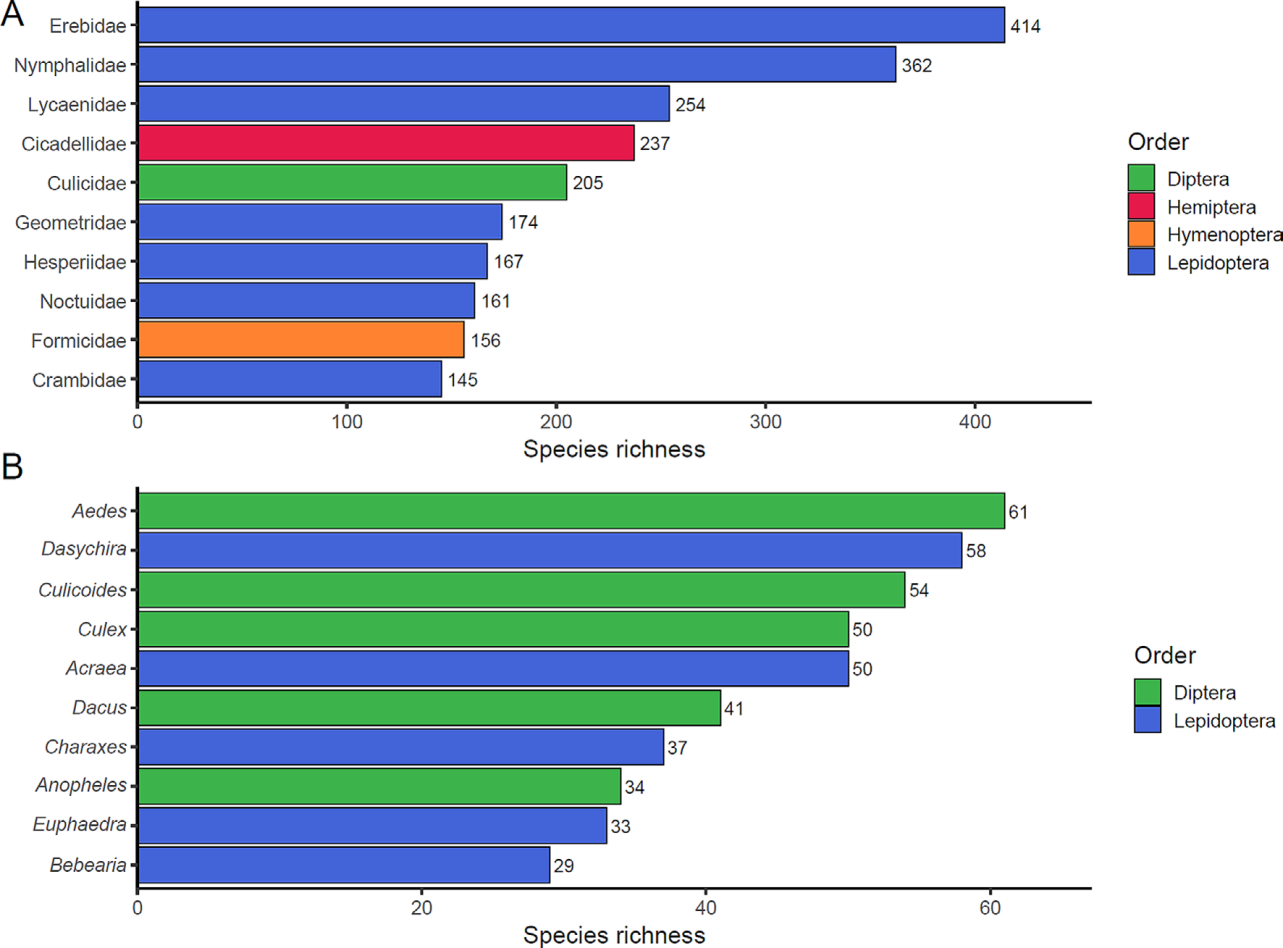
Ten most species‐rich insect families (A) and genera (B) in Nigeria according to GBIF records.

**FIGURE 2:**
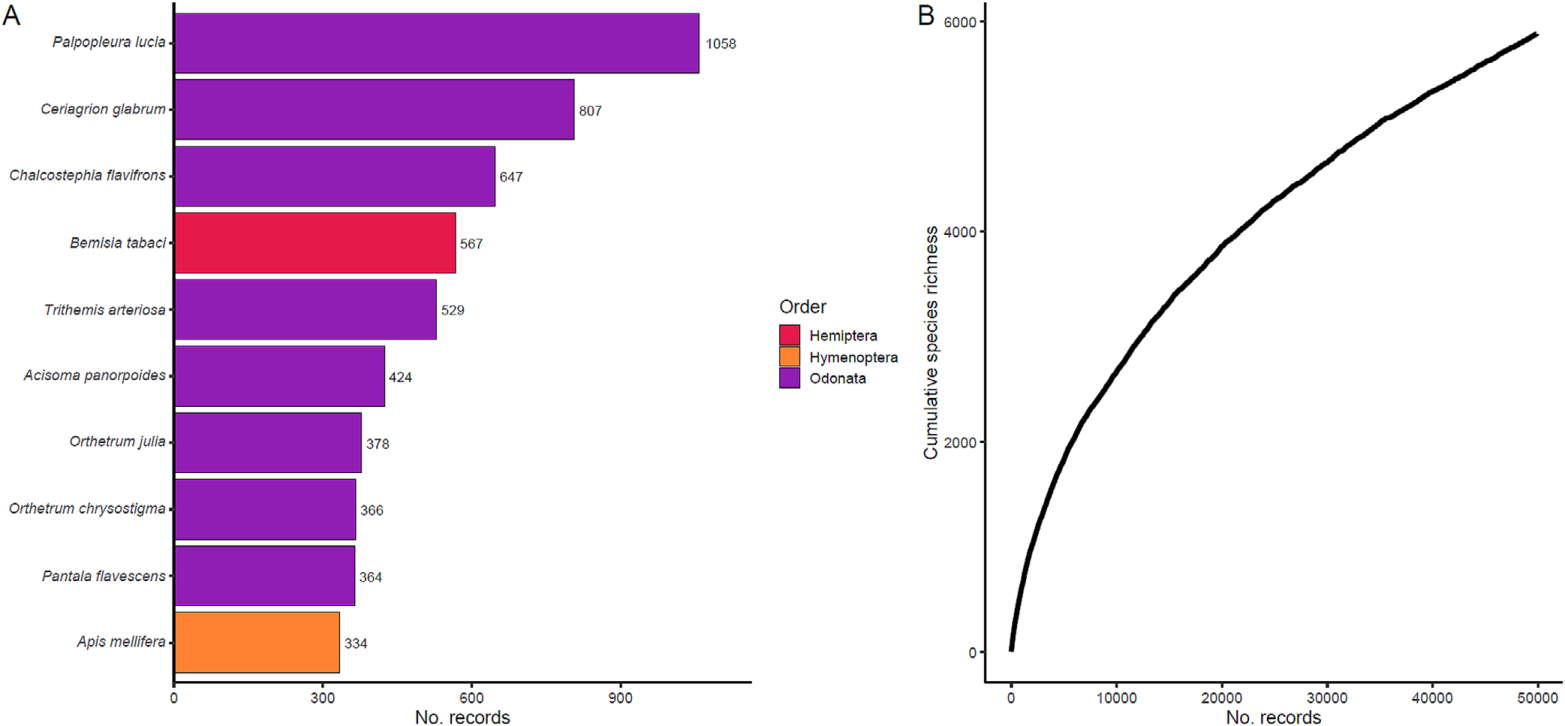
Ten insect species with the highest number of occurrence records in GBIF (A) and the relationship between GBIF records and cumulative insect species richness (B).

There were 19 orders, 305 families, 2515 genera and 5880 species from 49,854 occurrence records. Hemiptera had the highest number of families and contributed ~20% to the total number of families. However, Lepidoptera was the most dominant in terms of total number of genera, species and records, contributing ~40%, ~42% and ~37%, respectively (Table 1). At the family level, Erebidae (Lepidoptera) had the highest number of species, and together with nine other families (mostly of the lepidopteran order) accounted for nearly ~40% of all species (Figure 1A). At the genus level, *Aedes* (Diptera) had the highest number of species, but the top 10 genera were equally shared between Diptera and Lepidoptera (Figure 1B). The top 10 species with the highest number of records were dominated by Odonata (Figure 2A), with the most recorded species being *Palpopleura lucia*, contributing ~18% to the total number of species records. The least represented insects are members of the order Phasmida (stick insects), with one species (*Carausius morosus*) and one family, Lonchodidae.

**TABLE 1:** Diversity of insects in Nigeria according to GBIF records.

Insect order	Families	Genera	Species	Records
Lepidoptera	40	1008	2482	18,616
Diptera	54	265	949	10,094
Hemiptera	60	422	772	4434
Hymenoptera	31	192	494	2950
Coleoptera	35	257	483	2403
Odonata	14	85	296	9102
Orthoptera	8	87	100	583
Thysanoptera	3	48	76	993
Psocodea	18	38	60	208
Blattodea	6	33	59	139
Neuroptera	7	28	36	64
Trichoptera	6	15	23	75
Mantodea	7	14	19	45
Ephemeroptera	7	11	15	68
Mecoptera	1	2	4	40
Plecoptera	2	2	4	18
Siphonaptera	2	4	4	17
Dermaptera	3	3	3	4
Phasmida	1	1	1	1

**FIGURE 3:**
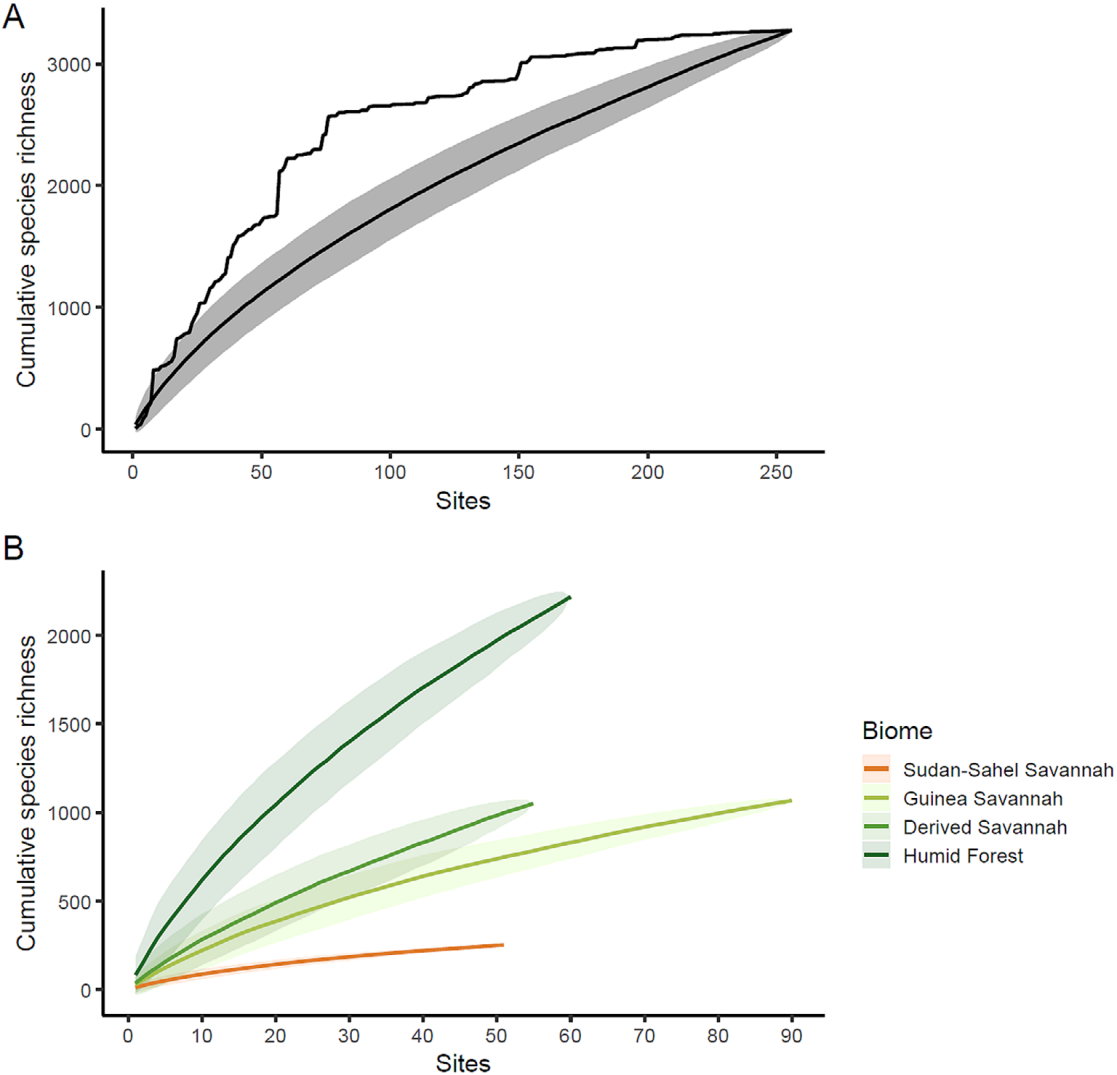
Species accumulation curves. (A) National. (B) By biomes. Rarefied curves (smooth lines and faded grey areas) are plotted from means and standard deviations of 1000 permutations of occurrence sites in random order. Collector curves (wrinkled line on national) show raw accumulated richness. Sites represent 0.5° × 0.5° grid cells.

**FIGURE 4:**
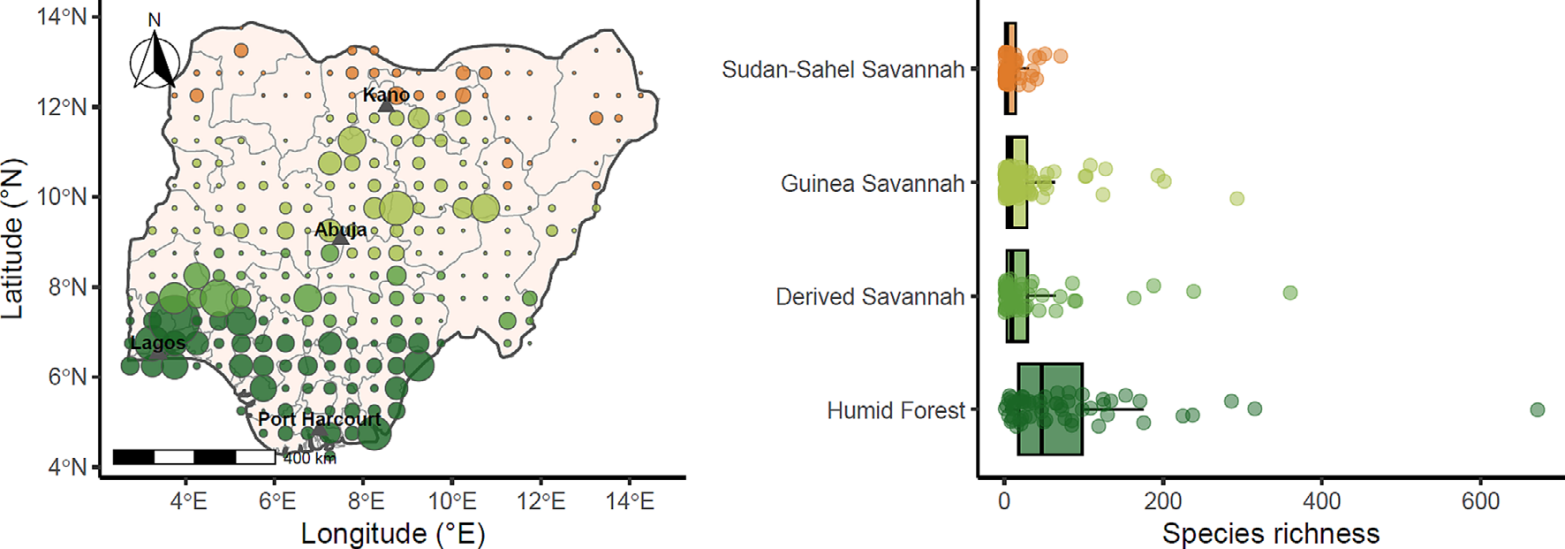
Species richness between Nigerian biomes according to GBIF records. Sites represent 0.5° × 0.5° grid cells. The upper boundary of the boxplots represents the 75th percentile, while the lower boundary represents the 25th percentile; the vertical line inside the box is the median.

Overall, species richness seems to increase cumulatively with the number of GBIF records (Figure 2B); on average, about eight occurrence records are needed to find a new species. As indicated by the steep accumulation curve, new species are discovered as sampling (or occurrence) sites increase (Figure 3A). Species richness estimators for the country, irrespective of biomes, indicate that ‘true’ richness is around 28,084. At the biome level, the Humid Forest had the highest richness while the Sudan‐Sahel Savannah had the lowest. However, in concomitance with the observation at the country level, not all species have been discovered in the biomes (Table 2); chances of finding new species are seemingly higher in the Humid Forest, Derived Savannah and Guinea Savannah than in the Sudan‐Sahel Savannah (Figure 3B). Overall, richness was, on average, higher in the southern part of Nigeria than in the north (Figure 4).

**TABLE 2:** Estimated species richness in Nigeria's major biomes according to GBIF records.

	Sites	Observed richness	Estimated richness (Chao 2)
Nigeria (as a whole)	256	3275	28,084
Sudan‐Sahel Savannah	51	251	1388
Guinea Savannah	90	1067	6443
Derived Savannah	55	1050	7908
Humid Forest	60	2217	9594

*Note:* Calculations are from geo‐referenced records that were assigned to 0.5° × 0.5° grid cells (sites).

## 4 Discussion

GBIF records are known to be biased by uneven sampling efforts; therefore, all insects in Nigeria may not be fully represented (Beck et al. 2013). This is important to consider because certain taxa may be systematically underrepresented, and the resulting analyses may reflect sampling effort rather than true diversity (Hortal et al. 2008; Rocchini et al. 2011). This is slightly evident in our study, where we observed stronger representation bias towards the southern part of Nigeria and towards charismatic, easily observable insects, especially at the order level and in terms of the number of records. Even within the highly represented insect taxa, the numbers may be understated. For example, while lepidopteran diversity in our study is broadly consistent with reports from some countries in Eastern (Tujuba et al. 2019) and Central Africa (Dongmo et al. 2023), other databases suggest higher diversity (Sáfián and Siklósi 2026; De Prins and De Prins 2026), indicating gaps that GBIF alone cannot resolve. Additional data quality issues with the GBIF data included low‐precision or absent coordinates, records falling outside Nigeria's polygon, and incomplete species names, all of which required exclusion during analyses. Finally, we could not account for taxonomically uncertain or potentially misidentified records due to the scale of our dataset; this continues to be a problem with GBIF data (Zizka et al. 2020; Führding‐Potschkat et al. 2022). Despite these constraints, the GBIF remains the most comprehensive resource available for investigating insect diversity in Nigeria—at least for now until coordinated nation‐wide field surveys and collections are established.

### 4.1 Trends in Insect Diversity and Distribution in Nigeria

Globally, there are 29 insect orders with Coleoptera having the highest number of species, followed sequentially by Lepidoptera, Diptera, Hymenoptera and Hemiptera (Zhang 2013; Stork 2018). Coleoptera is considered the most successful insect order due to a combination of factors, including (but not limited to) adaptive radiation, wide habitat niches, various sizes and feeding preferences (Bouchard et al. 2017). In this study, we found 19 orders representing ~66% of all known insect orders, slightly supporting the postulation of Scholtz and Mansell (2017) that all insect orders are found in Africa. However, in contrast to global reports (Zhang 2013; Stork 2018), Lepidoptera was the most dominant order in our study, surpassing Coleoptera with 1999 species. The reason for this trend is not apparent, but we speculate, firstly, that this could be due to bias from the GBIF data which includes records that may have more entries for ‘charismatic’ (or easily observable, collectable and identifiable) insects. The second speculation is that our observation could represent a ‘true’ trend for the country, given that Coleoptera (as the most diverse insect order) is already being challenged (Forbes et al. 2018). This, however, is our weakest speculation since GBIF records, while very extensive, do not represent all of Nigeria (see Appendices S1 and S2). Without systematic sampling, it may be difficult to draw a conclusion on what insect order is expected to be the most diverse in the country. Moreover, studies within Nigeria are also mixed with some reports highlighting the dominance of Lepidoptera (Abah et al. 2017; Naman and Abdullah 2019; Aina‐Oduntan et al. 2021), Diptera (Adu and Oyeniyi 2019; Membere et al. 2021), Coleoptera (Okrikata and Yusuf 2019) or Hymenoptera (Okeke et al. 2025; Abdullahi and Umar 2025). This is possibly due to different sampling techniques and intensities. Nonetheless, Lepidoptera, Diptera, Hemiptera, Hymenoptera and Coleoptera are all among the most diverse orders in this study.

At the family level, the lepidopteran families Erebidae and Nymphalidae were the most species‐rich. In South Africa, however, the family Geometridae was reported as the most diverse (Mecenero et al. 2022); nonetheless, this family is also among the top families in our study. Erebidae and Geometridae belong to the ‘moth’ group, which constitutes over 80% of all Lepidopterans, while Nymphalidae belongs to the butterfly group that constitutes ~10%. Moths and butterflies are known to differ mostly in their daily activity, with the former being nocturnal/crepuscular while the latter is diurnal (but see Kawahara et al. 2018). Our results mirror regional patterns in lepidopteran diversity (Tujuba et al. 2019) as well as those observed in Europe (Wiemers et al. 2018) and globally (Nieukerken et al. 2011; Goldstein 2017). Although the lepidopteran insects dominate the order and family level, the genus level presents a slightly different scenario where the dipteran genus (*Aedes*) is the most diverse, alongside other dipteran genera (*Culicoides*, *Culex*, *Dacus* and *Anopheles*) as well as some lepidopterans. Four of the most diverse dipteran genera (*Aedes, Culicoides, Culex and Anopheles*) are medically/veterinary important genera known to transmit vector‐borne diseases such as Yellow Fever, Bluetongue Virus, Lymphatic Filariasis and Malaria (Mike Service 2012).

*Carausius morosus* (Lonchodidae: Phasmida) is the least represented at the species, family and order level, as well as in records. It is a pet insect that is native to India but is currently considered an invasive species in many locations (see Krejsa 2025) so it is possible that it was introduced to Nigeria due to international travels. There is also a possibility of misidentification (discussed earlier) as this record was based on human observation, but a previous study has shown a similar observation for Nigeria (Krejsa 2025). Moreover, Phasmida is critically understudied in Nigeria and is one of the insect orders that require urgent attention in the country.

Species richness differed across biomes, being highest in the Humid Forest and lowest in the Sudan‐Sahel Savannah, seemingly following a latitudinal trend. This parallels assumptions about biodiversity patterns in which species diversity is suggested to decrease towards the poles (Macarthur 1965; Pianka 1966; Gaston 2000; Kinlock et al. 2018). This pattern is thought to be caused by several factors, including abiotic factors such as temperature (Rohde 1992, 1997) or precipitation (Meng et al. 2025), as well as energy/productivity (Currie et al. 1999), among others. The Humid Forest is characterised by higher precipitation, plant diversity and productivity (Sabatini et al. 2022), which has been shown to increase insect diversity (Schuldt et al. 2019). But whether the trend in our study reflects true biodiversity patterns or sampling bias (or both) remains to be investigated. Moreover, studies within the country have also reported mixed observations, where there is no consistent trend of higher richness in one biome than another (Abah et al. 2017; Yager et al. 2018; Okrikata and Yusuf 2019; Naman and Abdullah 2019; Adu and Oyeniyi 2019; Aina‐Oduntan et al. 2021; Okeke et al. 2025; Abdullahi and Umar 2025; Echor et al. 2025).

As shown in the species accumulation and estimation analyses, we are very far from the true insect diversity for the individual biomes as well as at the national level. Specifically, by simple fraction (5880 species from GBIF ÷ 28,084 estimated species) we report that only 21% of Nigeria's insect diversity is known while ~79% remains unknown. This exceeds current estimates of arthropod diversity in Nigeria (Federal Ministry of Environment 2001, 2025) as well as the last full checklist, which was done over four decades ago (see Medler 1980). In Afrotropical Africa, the most recent estimates for species richness are 975,179 (Stork 2018). By the GBIF records reported here, Nigeria's current total insect diversity is just a meagre fraction (< 1%) of the estimate for Afrotropical Africa. This, in addition to our species accumulation and estimator analysis, is an indication of how underrepresented and understudied the country is in terms of insects' diversity.

### 4.2 Problem(s) With Entomological Research in Nigeria

Entomological research in Nigeria is constrained by a set of deeply intertwined structural problems: persistent underfunding (Igiri et al. 2021; Igben and Etadafe 2023; Nnadieze and Oteyi 2023), limited institutional infrastructure (Akinwale 2010; Okafor 2024), nationwide insecurity (Duru and Asoegwu 2024; Adeodu et al. 2024), and poor prioritisation of scientific research (Alordiah et al. 2021; Igiri et al. 2021). All of these are a product of negligence at the state and federal levels of government (Ukaeje 2022; Ladi et al. 2025), which have been shown to affect biodiversity assessments/conservation in the country (Ijaiya et al. 2025). A direct consequence is the absence of locally and/or nationally coordinated systematic surveys and collections, the very foundations on which biodiversity research is built. And as we have reported here, nearly 80% of Nigeria's insect diversity remains undescribed or unrecorded, a figure that exceeds previous estimates of the arthropod diversity in the country (Medler 1980; Federal Ministry of Environment 2001, 2025). The socio‐economic conditions that have allowed this gap to persist must be addressed if any meaningful progress on entomological research in Nigeria is to be made. Until then, studies like ours will have to rely on digital databases, like the GBIF, which come with certain limitations (as discussed previously).

### 4.3 Future Directions

Given that digital records are probably the easiest to make, our first suggestion is the sensitisation of residents on the use of electronic devices to record and upload species occurrences to the GBIF or iNaturalist. However, as argued by Soriano‐Redondo et al. (2024), digital records should complement systematic surveys and identification, not replace them. More surveys and taxonomists are needed (Kehinde et al. 2014) to help establish local and national insect (or arthropod) collections. But all these will require extensive government support and funding, as well as the coordination/collaboration of researchers within and outside the country.

## 5 Conclusion

Insects are the most diverse and successful group of fauna on Earth (Eggleton 2020). However, country‐level assessment of their diversity is unknown for many countries in Africa. In the face of global insect threats and declines (IUCN 2026; Sánchez‐Bayo and Wyckhuys 2019), investigating insect diversity is essential for conserving biodiversity and preserving ecosystem functions. In this study, we investigated the diversity of Nigeria's insects and found 305 families and 5880 species, which only represent ~21% of their true diversity. Overall, the current state of Nigeria's insects reflects a dire situation that needs addressing through systematic surveys, as well as local and international collaboration, facilitated by extensive government support.

## Author Contributions

**Emelie Obi:** conceptualization (lead), data curation (lead), formal analysis (lead), investigation (lead), methodology (lead), writing – original draft (lead). **Mikaelison da Silva Lima:** methodology (supporting), validation (equal), writing – review and editing (equal). **Kelechi Amadi:** methodology (supporting), validation (equal), writing – review and editing (equal). **Maduamaka C. Abajue:** methodology (supporting), supervision (lead), validation (equal), writing – review and editing (equal).

## Funding

The authors have nothing to report.

## Conflicts of Interest

The authors declare no conflicts of interest.

## Supporting information


**Appendix S1:** Raw species richness (A) and records (B) by geo‐referenced points from GBIF.
**Appendix S2:** Cumulative species richness (A) and annual records (B) per year. Data calculated only from GBIF records with years specified.

## Data Availability

Data and R codes are available on Dryad (https://doi.org/10.5061/dryad.p5hqbzm37).
