# Supplementary material for: Diversity and Distribution Patterns of Insects in Nigeria: A GBIF‐Based Appraisal
Source: Ecol Evol. 2026 Jul 29;16(8):e74090. doi: 10.1002/ece3.74090 (PMC13417025; doi:10.1002/ece3.74090)
Supplement: Supplementary file 1 — Appendix S1: Raw species richness (A) and records (B) by geo‐referenced points from GBIF. Appendix S2: Cumulative species richness (A) and annual records (B) per year. Data calculated only from GBIF records with years specified. [file ECE3-16-e74090-s001.pdf]

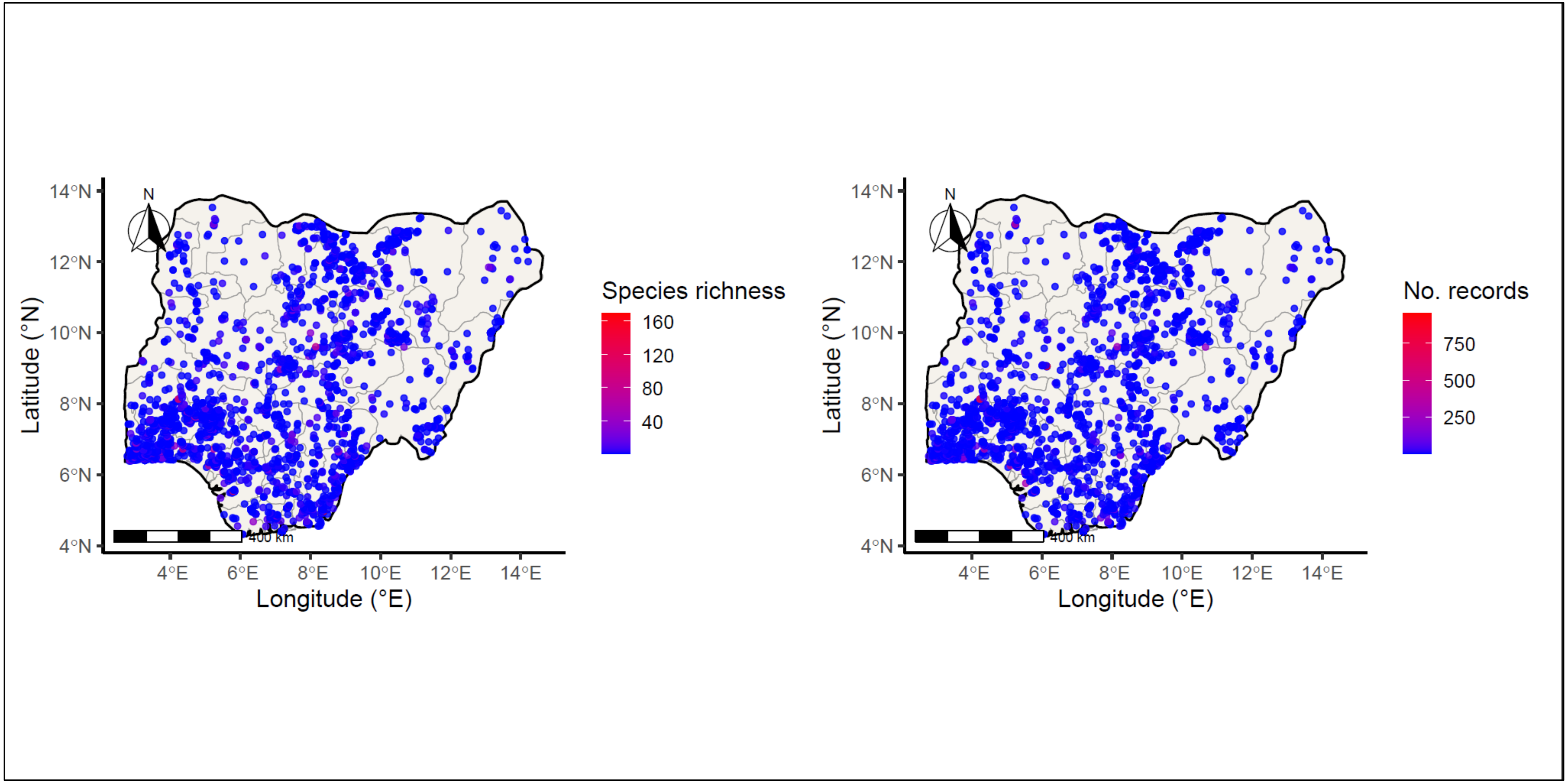

Appendix S1: Raw species richness (A) and records (B) by geo-referenced points from GBIF

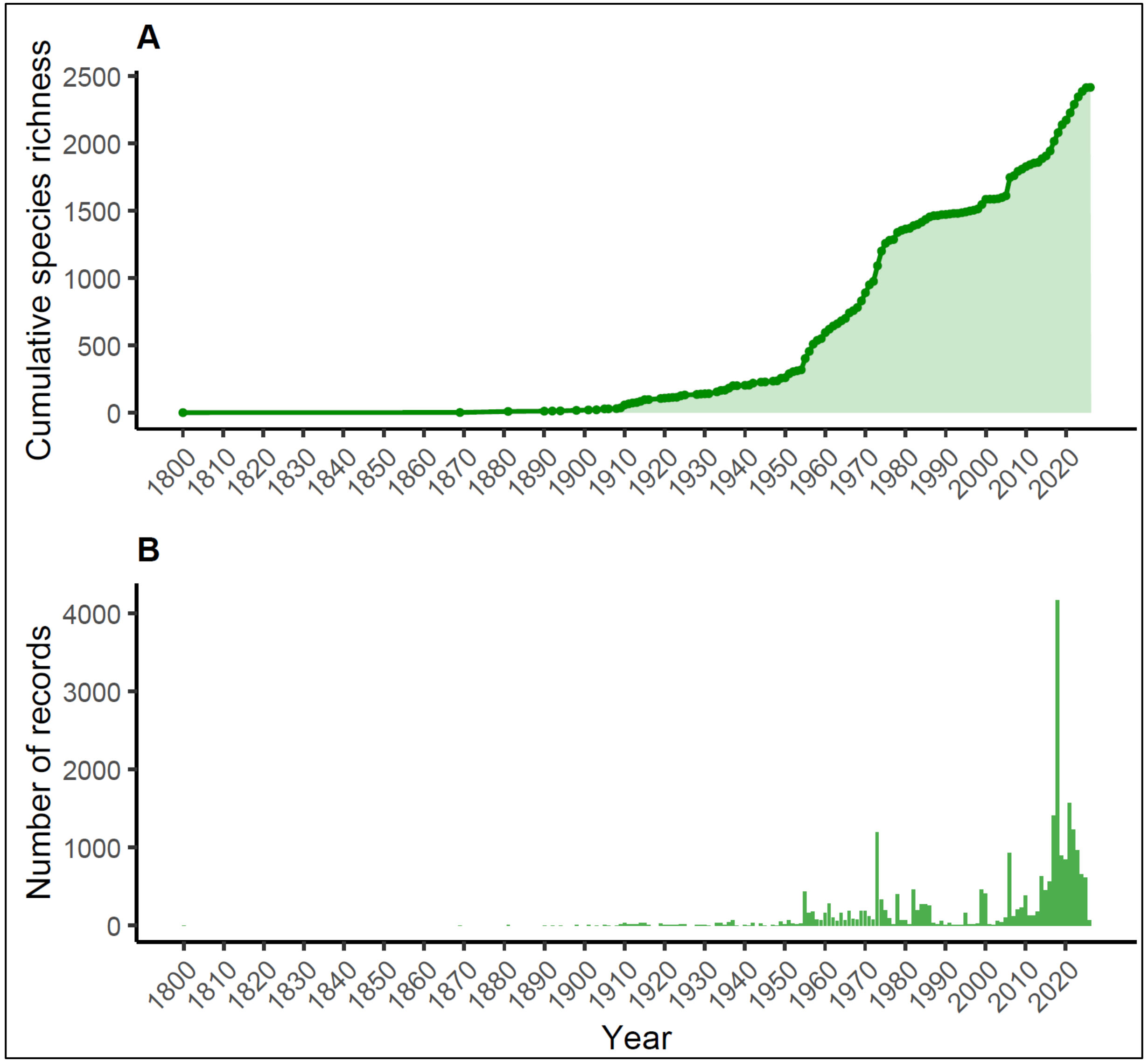

Appendix S2: Cumulative species richness (A) and annual records (B) per year. Data calculated only from GBIF records with years specified
